# Better In Vitro Tools for Exploring *Chlamydia trachomatis* Pathogenesis

**DOI:** 10.3390/life12071065

**Published:** 2022-07-15

**Authors:** Simone Filardo, Marisa Di Pietro, Rosa Sessa

**Affiliations:** Department of Public Health and Infectious Diseases, University of Rome “Sapienza”, 00185 Rome, Italy; marisa.dipietro@uniroma1.it (M.D.P.); rosa.sessa@uniroma1.it (R.S.)

**Keywords:** *Chlamydia trachomatis*, host–cell interaction, in vitro cell culture models

## Abstract

Currently, *Chlamydia trachomatis* still possesses a significant impact on public health, with more than 130 million new cases each year, alongside a high prevalence of asymptomatic infections (approximately 80% in women and 50% in men). *C. trachomatis* infection involves a wide range of different cell types, from cervical epithelial cells, testicular Sertoli cells to Synovial cells, leading to a broad spectrum of pathologies of varying severity both in women and in men. Several two-dimensional in vitro cellular models have been employed for investigating *C. trachomatis* host–cell interaction, although they present several limitations, such as the inability to mimic the complex and dynamically changing structure of in vivo human host-tissues. Here, we present a brief overview of the most cutting-edge three-dimensional cell-culture models that mimic the pathophysiology of in vivo human tissues and organs for better translating experimental findings into a clinical setting. Future perspectives in the field of *C. trachomatis* research are also provided.

## 1. Introduction

*Chlamydia trachomatis* is an obligate intracellular human pathogen responsible for a range of diseases of public health importance. Indeed, this pathogen is the leading cause of sexually transmitted bacterial infection worldwide, with more than 130 million new cases each year [1]; the prevalence and incidence estimates are highest for both women and men in the Western Pacific Region, and rates peak in the region of the Americas, although the real prevalence of *C. trachomatis* genital infection remains unknown and probably underestimated, due to the high proportion of asymptomatic infections [1].

In women, the most common clinical manifestations following *C. trachomatis* genital infection are mainly cervicitis and salpingitis, in men urethritis and epididymitis, and it is becoming increasingly accepted as a causative agent of prostatitis [2,3]. In more than 80% of women and 50% of men, *C. trachomatis* infection is asymptomatic and, hence, if left untreated, it eventually leads to several complications with serious consequences, including infertility and reactive arthritis [3,4]. Furthermore, *C. trachomatis* infection can also be transmitted to infants following the direct contact with infective cervical secretions during delivery, resulting in neonatal conjunctivitis and pneumonitis [5,6,7]. Lastly, there is evidence that *C. trachomatis* infection increases the risk of acquiring and transmitting human immunodeficiency virus by three to four times and, more recently, it has been associated with Human Papillomavirus-related cervical cancer [8,9].

Over the past decades, pathogenic mechanisms underlying *C. trachomatis* mediated complications have received significant research attention. Specifically, several in vitro cellular models, based on two-dimensional (2D) cultures of immortalized cells, have been employed for investigating *C. trachomatis* host–cell interaction, focusing on the characteristics of chlamydial developmental cycle and its intracellular survival strategies. These systems have been very useful due to their highly controlled experimental conditions, although they fail to mimic the complex and dynamically changing structure of in vivo human host-tissues [10,11]. As a way to overcome these issues, in vivo animal models, mainly mice, have been used to elucidate the natural history of the disease, the pathogenetic mechanisms underlying chlamydial infection and its chronic outcomes, as well as for drug and vaccine development; however, animal models possess important differences from the human host in the clearance of chlamydial infection and in the defense immune mechanisms [12,13]. Additionally, in vivo studies raise important ethical concerns, and, hence, better alternatives are needed [12].

Here, we present a brief overview of the most cutting-edge cell-culture models that mimic the pathophysiology of in vivo human tissues and organs for translating experimental findings into a clinical setting. Future perspectives in the field of *C. trachomatis* research are also provided.

## 2. In Vitro Modeling of *C. trachomatis* Infection

Cell-culture monolayers have been used for isolating *C. trachomatis* from clinical specimens, for studying chlamydial biology, virulence factors, molecular and cellular pathways, or for drug screening [14,15,16,17,18,19,20,21]. In particular, the most widely used models in the history of *C. trachomatis* research are 2D in vitro cell-culture models based on immortalized cells, including mostly HeLa and McCoy cells, as well as other cell lines like HEC-1B, CaCo2, HEp2, and monocyte-macrophages [14,15,16,17,18,19,20,21,22,23,24].

Unlike most bacteria, *C. trachomatis* developmental cycle occurs entirely within a cell-derived membrane bound vesicle where it undergoes dramatic physiological and morphological changes, alternating between two functionally distinct forms: the elementary body (EB) and the reticulate body (RB). Chlamydial EB is the small (200 nm) extracellular and infectious form, characterized by minimal metabolic activity [25,26]. In contrast, chlamydial RB is the large (800 nm) and metabolically active form, responsible for intracellular replication [27].

Soon after attachment to the host cell, EBs are internalized and confined to a parasitophorous vacuole termed inclusion, through a process requiring the secretion of Type III secretion system (T3SS) effector proteins, such as the translocated actin-recruiting phosphoprotein (TarP), the translocated early phosphoprotein (TepP) and inclusion membrane (Inc) proteins [28]. Specifically, TarP rapidly recruits actin to the entry site in order to exploit the host cytoskeleton and facilitate entry of chlamydial EBs, whereas TepP and Incs are essential for regulating the host immune response to *C. trachomatis*, allowing its survival within the host cell [29].

Once inside the inclusion vacuole, the EBs differentiate to RBs, which replicate by binary fission within 24 h post-infection [30]. Approximately 24–36 h post-infection, the majority of RBs begin to transition back to EBs [30]; and at about 36–48 h post-infection, the EBs are finally released from the host by cell lysis or through a mechanism called extrusion, and a multitude of infectious EBs spread and infect neighboring cells [31].

In order to maintain its intracellular survival, *C. trachomatis* has adopted a variety of strategies. For example, anti-oxidative stress mechanisms by which *C. trachomatis* may resist the host clearance, include the suppression of NADPH oxidase activity and activation of superoxide dismutase [32,33]. Additionally, inhibition of pro-apoptotic pathways and activation of pro-survival pathways have been reportedly employed by *C. trachomatis* in the early stage of growth cycle [34]. A further survival strategy of *C. trachomatis* is the development of persistent forms. Indeed, under stressful conditions (e.g., antibiotic or IFN-γ treatment, iron deficiency, coinfection with HSV-2, etc.), *C. trachomatis* has been shown to stop its developmental cycle, generating persistent forms that remain inside the host cell for a long time due to their ability to evade the immune system, leading to a chronic inflammatory state responsible for the tissue damage [35,36]. More recently, it has been demonstrated that the development of *C. trachomatis* persistent forms may be related to T3SS dysfunction and subsequent blocked secretion of bacterial effector proteins, resulting in an enhanced evasion of both extracellular and intracellular host defenses [37].

Recently, clinically relevant advances in the knowledge of the pathogenetic mechanisms associated to *C. trachomatis* genital infection have been provided by in vitro 2D cell-culture models based on primary human cells. For example, primary polarized human ecto- and endo-cervical explants have been used to demonstrate the ability of *C. trachomatis* to alter epithelial structure by inducing epithelial to mesenchymal transition [38], a process known to contribute pathologically to fibrosis and cancer progression [39]. A more comprehensive in vitro model of fallopian tube based on primary human polarized multiciliated epithelial cells, producing mucin, has allowed to better characterize the cell response to *C. trachomatis* infection [40].

Interesting 2D in vitro models based on human primary Sertoli and prostate epithelial cells have highlighted potential cellular and molecular mechanisms underlying male infertility; indeed, the prostate might be a trojan horse for *C. trachomatis* infection of the reproductive tract, from where this pathogen may disseminate in the host, reaching the testis [41]. Once inside Sertoli cells, located within the seminiferous tubule and responsible for protective functions toward germ cells, *C. trachomatis* might damage the Sertoli cell barrier function, and, hence, the spermatogenesis [42,43].

Lastly, a primary human synovial cell model, known to be involved in the reactive arthritis following chlamydial genital infection, has evidenced another escape strategy of *C. trachomatis* from host cell defense pathways, through a dysregulated inflammasome activation [44,45].

## 3. Current Advances in Three-Dimensional Cell-Culture Modeling

Three-dimensional (3D) cell-culture models based on primary cells are acquiring great importance as a new and robust platform for studying complex biological processes and might be a promising alternative in *C. trachomatis* pathogenetic studies [13]. In this regard, 3D models might help in recreating the microenvironment that *C. trachomatis* encounters in the host tissue, allowing a deeper understanding of host–pathogen interactions since these systems promote direct cell-to-cell contact, interactions of cells with the extracellular matrix and in vivo like exchange of soluble factors [10,11]. Furthermore, 3D cell culture models are known to retain the cellular structure and spatial orientation more closely resembling the in vivo parental tissue than the more widespread 2D cell culture models [10,11].

In the literature, several approaches have been developed for generating 3D models (Table 1), including scaffold-based 3D cultures, that use matrices for cells adhesion, and non-scaffold 3D cultures, that, by contrast, lead to cell assembly into spheroids. The non-scaffold 3D cultures promote cell-to-cell rather than cell-to-extra cellular matrix interactions, favoring the natural aggregation and assembly of cells in spheroids that better mimic the in vivo organ formations, hence the name organoids [10,11,46].

Amongst the different methodologies, 3D bioprinting technologies have opened a completely new field for tissue engineering [47]. Several approaches are available, such as filament deposition modelling or stereolitography printing aided by computer assisted design (CAD) models or medical imaging data, which can be used to assemble cells, extracellular matrix proteins, cytokines, growth factors and other components into a bio-inspired tissue structure [48]. After the 3D tissue model is printed, the fabricated tissue changes over time due to cell self-organization and differentiation under different environmental stimuli, just like the in vivo tissue [49].

More recently, a novel cutting-edge technology has been introduced, namely the organ-on-a-chip (OOAC) [50]. OOAC recreates the complex and dynamic operations occurring in human tissues for real time monitoring of the cell and tissue response to an infection [50]. In particular, miniaturized cell-culture micro-environments with microchannels and chambers mimic the human cell pathophysiological environment, allowing the high throughput screening with the integration of automation and smart analysis systems [51,52]. OOAC can incorporate multiple cell layers, mimicking the complex cell interactions that occur in in vivo human tissues, and multiple organs can also be connected [53]. Moreover, several actuators and sensors can be integrated for various analysis, potentially providing more precise and relevant clinical data [50,51]. Lastly, they can incorporate various biomaterials for the fabrication of cells’ microenvironment (polydimethylsiloxane, polymethylmethacrylate, polystyrene, etc.) [50].

Notwithstanding the different 3D cell-culture models, to date, few of them have been utilized in chlamydial research. Amongst them, recent *C. trachomatis* studies have explored human organoids [13], produced by embedding primary cell cultures into Matrigel-based scaffold, an animal-derived extra-cellular matrix widely used for organoid cultures [54]. The first model described in literature is endometrial organoid, generated from murine primary cells [55,56]. The second model to be explored has been ectocervical organoid from primary human cells, where the development of *C. trachomatis* infection was observed, as well as the potential contribution of this pathogen to neoplastic progression in presence of HPV infection [57]. Lastly, primary human fallopian tube organoid was exploited to establish a model of *C. trachomatis* chronic infection, in order to study the long-term changes of the epithelium potentially involved in tubal pathologies [58].

**Table 1:** Characteristics of the main technologies for the fabrication of advanced 3D cell-culture models.

Scaffold-Based 3D Cell-Culture Models			
Technology	General Characteristics	Advantages	Disadvantages
**Hydrogels/Matrigels** [54]	3D hydrophilic extracellular matrix-rich meshes used as framework to surround and encapsulate cells	hydrophilic nature, chemical stability, biological compatibility, and biodegradability	labor intensive, high variability in matrix composition, long working time
**Non-scaffold-based 3D cell-culture models**			
**Bioreactors** [59]	3D spheroids generated by creating a micro-gravity environment via rotational motion	limited cell damage and long-term culture periods due to low-shear environment, enhanced natural diffusion of gas and nutrients	heterogeneous spheroid size, challenging to monitor
**Spinner Flasks** [60]	spontaneous cell collision and adhesion in cell suspension via continuous rotary motion	enhanced gas and nutrient diffusion, large number of spheroids	harmful shear stress forces, challenging to monitor
**Hanging drops** [61]	single spheroid per droplet via cell self-aggregation following upside-down incubation of droplets	basic laboratory equipment, easy to monitor	limited number of spheroids, long working time
**Ultra-low attachment plates** [62]	cell suspension loaded on round-bottom cell culture microplates covered with non-adhesive materials	inexpensive and easy to use, spheroid size and shape reproducible and homogenous, high throughput screening, easy to monitor	limited number of spheroids, incompatible for large spheroids
**Centrifugation pellet cultures** [63]	cell aggregation via centrifugation of cell suspension	inexpensive and easy to use, large number of spheroids	harmful shear stress forces, challenging to monitor
**Electric, magnetic and ultrasound based cultures** [63,64,65]	spheroid formation via electric or magnetic fields, or ultrasound forces	control of spheroid’s development settings	challenges in controlling spheroid size, specific equipment, harmful external forces
**Microwell arrays** [66]	cell suspensions loaded in microwells layered with non-adhesive substances via micro-patterning	inexpensive, easy to use, spheroid size and shape reproducible and homogenous, complex-shaped spheroids, high throughput screening as well as standard monitoring methodologies	incompatible with large spheroids
**Microfluidic systems** [67]	cell suspensions loaded through a micro-channel system in microwells, leading to cell aggregation via small bioreactors	easy to use and fast, enhanced natural diffusion of gas and nutrients, large number of spheroids with homogenous size, high throughput screening	advanced specialized laboratories
**3D Bioprinting** [48,49]	tissue-like structure formation by automated deposition of cells, biological materials, and supportive matrix in layers	possibility to precisely arrange cells, enhanced cell viability, functions, migration, and self-assembly, high throughput screening	harmful shear stress forces, expensive, long working time

## 4. Discussion

*C. trachomatis* still possesses a significant impact on public health, for the high prevalence of asymptomatic infections and its ability to involve a wide range of different cell types, from cervical epithelial cells, testicular Sertoli cells to Synovial cells, leading to a broad spectrum of pathologies of varying severity both in women and in men [3,68]. Consequently, there is increasing research interest in *C. trachomatis*; this is also due to the emergence of important breakthroughs in the genetic manipulation of chlamydial EBs, as well as the introduction of advanced molecular techniques for DNA sequencing, for exploring the diverse defense factors of the genital tract [69,70].

Two-dimensional in vitro models, over the years, have paved the road towards the understanding of *C. trachomatis* genital infection and outcomes. To date, alternative and more advanced platforms, like 3D cell cultures, that provide increased similarity to the in vivo physiology and pathology, have been helpful for investigating the complex pathogenetic mechanisms of other infectious pathogens, like SARS-CoV-2, and might also be fundamental for studying *C. trachomatis* genital infection, although their adoption is still at an early stage [55,56,71].

Several systems exist as above described, and all of them have significantly expanded different aspects of biomedical research since they can mimic various important functions of different organs and tissues in vitro, or even replicate entire organs, and, hence, constitute a more realistic approach relative to traditional cell culture models. Indeed, they can incorporate multiple different structures and cell types, as for example immune cells, like monocyte-macrophages and neutrophils, or they can be used for studying co-infections, providing deeper insights into cellular interactions, drug screening, and the pathophysiology of various diseases [13]. However, they present several critical issues, including the lack of affordability, the more stringent culture conditions, low reproducibility, as well as the extensive operator’s skills and dedicated advanced facilities. In this regard, for example, 3D bioprinting is undermined by the difficulty in maintaining cell viability and function beyond diffusion limits, since the transport of nutrients and oxygen to each cell, as well as the removal of waste, must be guaranteed [48,49]. OOAC systems are still in development because they present difficulties in completely replicating the entire physiology of an organ, the need for better biocompatible materials with improved performance for cell environment fabrication, as well as the limited sensitivity and specificity of available sensors for monitoring physiological parameters more accurately [50,53]. Moreover, the complexity of these models is increased when multiple organs or complex tissues are integrated on a chip and a large amount of data is produced, requiring high-throughput analysis to reach accurate conclusions, like machine-learning techniques [52].

In the future, these advanced technologies for mimicking the physiology of human tissues and organs will represent a robust platform with the potentiality to lead to important breakthrough in the fields of chlamydial pathogenesis, diagnosis, prevention, and treatment.

## Data Availability

Not applicable.
